# Evaluation of Fatty Acid Distributions and Triacylglycerol Species in Sow Milk and Commercial Piglet Formulas: A Comparative Study Based on Fat Sources and Lactation Stages

**DOI:** 10.3390/ani13010124

**Published:** 2022-12-28

**Authors:** Cuirong Ren, Jun Jin, Thom Huppertz, Yanbing Zhang, Qingzhe Jin, Xingguo Wang

**Affiliations:** 1 State Key Laboratory of Food Science and Technology, Collaborative Innovation Center of Food Safety and Quality Control in Jiangsu Province, International Joint Research Laboratory on Lipid Nutrition and Safety School of Food Science and Technology, Jiangnan University, Wuxi 214122, China; 2 Food Quality & Design Group, Wageningen University and Research, 6708WG Wageningen, The Netherlands; 3 FrieslandCampina, 3818LE Amersfoort, The Netherlands; 4 HuaNong Lipid Nutrition Technology Co., Ltd. in Shandong Province, Binzhou 256600, China

**Keywords:** fat, sow milk, piglet formula, *sn*-2 fatty acid, triacylglycerol

## Abstract

**Simple Summary:**

Fat ingredients used in piglet formulas differ from sow milk fats in fatty acid compositions and triacylglycerol (TAG) species, and therefore may be less optimal for piglet growth. It is necessary to well-define fat characteristics (including fatty acid distributions and triacylglycerol species) of sow milk in a sufficient number of samples, as well as lipid profiles of piglet formulas. Total fatty acid and *sn*-2 fatty acid compositions, and triacylglycerol species in sow colostrum, sow milk, and piglet formulas were analyzed in the present study. Colostrum, milk, and piglet formula samples were notably distinguished into three groups based on their fatty acids and triacylglycerols, among which triacylglycerols were the most differentiated index. The results are beneficial for providing inspirations for production of sow milk fat equivalents.

**Abstract:**

Total fatty acid and *sn*-2 fatty acid compositions, and triacylglycerol (TAG) species in 130 sow colostrum, 100 sow milk, and 22 piglet formula samples were analyzed in the present study. Significant differences were found in concentrations of medium chain-saturated fatty acids (MC-SFAs) and distributions of palmitic acid (P) and oleic (O)/linoleic (L) acid. The levels of MC-SFAs in sow colostrum and sow milk fats (2.4–3.1%) were significantly lower than those in piglet formulas (7.9–27.2%). Approximately 63% of palmitic acid was located at the *sn*-2 position in both sow colostrum and milk fats, which was significantly higher than in piglet formula fats (21.1–39.1%). Correspondingly, only 17.8–28.3% of oleic and linoleic acids were at the *sn*-2 position in sow milk fats, contributing to their typical triacylglycerol structure in sow colostrum and milk, whose palmitic acid connected to the *sn*-2 position and unsaturated fatty acids located at the *sn*-1,3 positions. Sow colostrum, milk, and piglet formulas were notably distinguished into three groups based on their fatty acids and TAGs, among which triacylglycerols were the most differentiated index. A total of 51 TAG species (including their isomers) differed significantly between sow colostrum and milk and piglet formulas. OPL and OPO were the most important differentiating TAGs. The large amount of sn-2 esterified palmitic acid plays a key role in improving the absorption of fat and calcium. The results provide suggestions for design of sow milk fat equivalents.

## 1. Introduction

The major goals in modern pig breeding during the last few decades have been to increase prolificacy and carcass merit [1,2]. However, increased preweaning mortality of piglets results in undesirable economic losses and limitations in pork supply [3]. Preweaning mortalities have varied widely among different countries, e.g., 9% in Brazil, but 14% in Canada and Denmark [4]. In order to ensure piglet survival and growth, sow colostrum and milk are crucial, with milk fat serving as a significant energy source and providing essential fatty acids for the development of newborns [5,6]. Although sow milk fat accounts for only 5–8% of sow milk mass, its digestibility is >98% and it provides piglets with about 50% of the energy [7,8]. Insufficient intake of sow milk and milk fat contributes to preweaning mortality. Therefore, additional milk and milk fat, such as pig milk powders, are suggested to be included into diets for piglets during the pre-weaning period, to improve their survival and growth.

However, commercial piglet formulas are primarily concerned with meeting energy requirements rather than nutrients, including specific fat components [9]. Fat ingredients used in piglet formulas differ from sow milk fat in terms of fatty acid composition and triacylglycerol (TAG) species, and therefore may be less suitable for piglet growth. TAGs are the main component (about 98%) in sow milk fat, which have special fatty acid compositions and unique distributions [10]. The major fatty acids in sow milk fat are oleic acid (O, 25.1–30.9%), palmitic acid (P, 21.1–24.8%) and linoleic acid (L, 23.7–38.3%) [11]. Unsaturated fatty acids (UFAs), such as oleic acid and linoleic acid, show considerable *sn*-1,3 positional selectivity, whereas saturated fatty acids (SFAs), especially palmitic acid, are typically found at the *sn*-2 position of TAG molecules [12,13,14]. In our previous study, the most prevalent TAG types in sow milk fat, accounting for ~50% of TAGs, were USU TAGs (i.e., TAGs with one saturated fatty acid at the *sn*-2 position and two unsaturated fatty acids at the *sn*-1,3 positions), mainly OPL, LPL, and OPO [15]. Such a unique structure has a great effect on the TAG digestion and absorption [16,17,18]. Previously, we have improved the knowledge on the TAG composition of sow milk through the evaluation of the effects of different sow breeds and lactation stages (colostrum and milk) on the composition of TAG, but did not include positional distribution of fatty acids. Growing evidence demonstrates the positional distribution of fatty acids has been recognized as the important factor of dietary fat digestion and absorption [19,20]. For example, the high level of *sn*-2 palmitic acid plays a key role in enhancing the absorption of fatty acids and calcium [21]. Hence, understanding of positional distribution of fatty acids in sow milk and piglet formulas is likely a critical nutritional factor. However, very few studies thus far focused on studying and comparing the differences in not only fatty acid composition, but also positional distribution of fatty acids, and triacyl–glycerol species, and provide a direct comparison between commercial piglet formulas and sow milk. In addition, fatty acid composition of sow milk has obvious regional variations, which was consistent with the previous studies that fatty acids composition of the milk changes with the dietary fat sources [22,23]. Therefore, regional dietary characteristics should be considered when developing commercial formulas of piglets for specific geographies, e.g., China. In this regard, the current commercial formulas may have further room for optimization for piglets.

Although compositional differences between sow milk and piglet formula have been revealed for a small number of samples (sow milk, *n* = 6; formula, *n* = 3) obtained from Europe [24] and Canada [12], no studies comprehensively evaluated and compared the fat components (especially the fatty acids and their distributions on triacylglycerol molecules) of both sow milks and formulas on the Chinese market in an adequate sample size. It is necessary to properly define fat characteristics (including fatty acid distribution and triacylglycerol species) of sow milks in a sufficient number of samples collected from farms in China, as well as lipid profiles of piglet formulas on Chinese market. The aim of this study was therefore to improve the knowledge on the lipid composition of sow milk and sow colostrum, as well as commercial piglet formulas, and establish the foundation for the production of sow milk fat substitutes, by focusing not only on fatty acid composition, but also on positional distribution of the fatty acids. The results can form an important basis for further optimization of the fat blends used in piglet formulas.

## 2. Materials and Methods

### 2.1. Sampling and Chemicals

A total of 130 sow colostrum samples and 100 sow milk samples were collected from healthy sows in China. Colostrum (15 mL) was collected on the first day after the birth of the first piglet by hand-milking. Milk (15 mL) was collected on the 15th day of lactation after injecting 2 mL of oxytocin. The basal diet of all sows was composed of corn-soybean to meet the nutrient requirements for pregnant and lactating sows, as recommended [25]. The collected samples were transported to the laboratory on ice and stored at −80 °C until analysis. A total of 22 commercial piglet formulas, produced by local manufactures, were purchased from the Chinese market. J&K Scientific supplied HPLC-grade methanol and n-hexane (Beijing, China). Sigma-Aldrich Chemicals Co., Ltd. (Shanghai, China) supplied a standard mixture of fatty acid methyl esters, thin-layer chromatography plates (silica gel 60, 10 × 20 cm, 0.20–0.50 mm), petroleum ether, ethanol, diethyl ether, ammonia solution, and other analytical-grade reagents.

### 2.2. Fat Extraction from Sow Milks and Piglet Formulas

The Röse–Gottlieb method, as previously described [26], was used to extract fat from sow milks and commercial piglet formulae.

### 2.3. Analysis of Fatty Acid Composition

Typically, 2 mL of n-hexane and 1 mL of KOH-CH_3_OH (2 M) were added to 20 mg of each fat sample. After shaking for 2 min, the appropriate amount of Na_2_SO_4_ was then added to finish saponification. The analysis of fatty acid composition was determined based on our previous work [11]. The Agilent 7820A (Agilent, Palo Alto, CA, USA) equipped with a hydrogen flame ionization detector and a Trace TR-FAME capillary column (60 m × 0.25 mm × 0.25 μm, Thermo Fisher, Waltham, MA, USA) was used to examine the sample, which included the fatty acid methyl esters. The employed program for determination was firstly maintained at 60 °C for 3 min, then increased at 5 °C min^−1^ to 175 °C and held at 175 °C for 15 min, followed by increasing at 2 °C min^−1^ to 220 °C (held for 10 min). The injector and detector temperatures were both set to 250 °C. The carrier gas was nitrogen (1.0 mL/min), and the split ratio was set to 1:100. The retention times of the standards were used to identify fatty acids.

### 2.4. Analysis of sn-2 Fatty Acids

Fatty acid compositions of the 2-monoacylglycerol (2-MAG) were determined according to the methods as described by [27]. Tris buffer (pH 7.6, 8 mL), bile salts (0.05%, 2 mL), and calcium chloride (2.2%, 0.8 mL) were added into a centrifuge tube containing 50 mg of the fat sample. Porcine pancreatic lipase (50 mg) was added to hydrolyze fatty acids distributed at the *sn*-1,3 positions in a 37 °C water bath for 3 min. After extracting the hydrolytic products with 2 mL of diethyl ether, they were separated on a silica gel plate with hexane, diethyl ether, and acetic acid (50:50:1, *v*/*v*/*v*). The band corresponding to 2-monoacylglycerol (2-MAG) was then isolated and extracted with 1 mL of diethyl ether.

### 2.5. Determination of Triacylglycerol Composition

TAG composition of each fat sample was analyzed using an ultra-performance liquid chromatography coupled to a quadrupole time-of-flight mass spectrometry system (UPLC-Q-TOF-MS; Waters, Milford, MA, USA) according to the method reported previously [28,29].

### 2.6. Statistical Analysis

All measurements were taken in triplicate and the findings were expressed as average values ± standard deviation (AVE ± SD). The data was analyzed using IBM SPSS 22.0 to find significant differences. SIMCA (Version 14.1, MKS Umetrics, Malmo, Sweden) was used to perform principal component analysis (PCA) analyses. A PERMANOVA test was performed to determine the compositional differences among test groups.

## 3. Results

### 3.1. Fat Characteristics of Piglet Formulas

**Figure 1:**
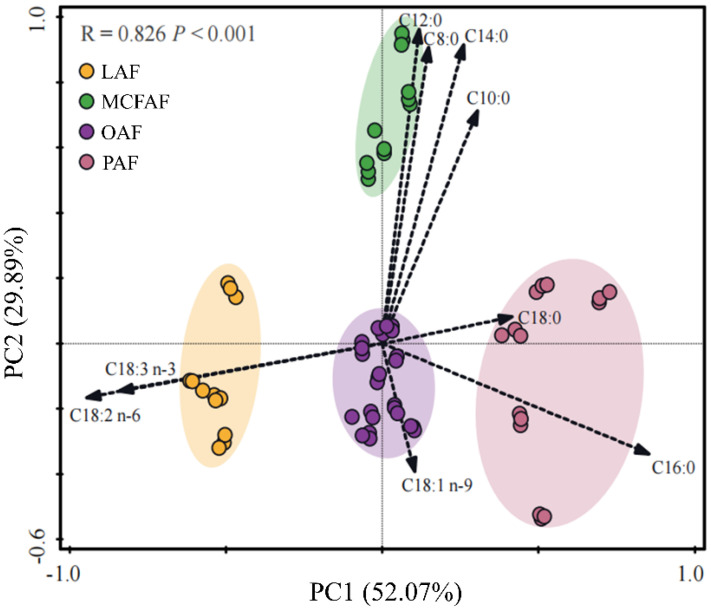
Principal component analysis of fatty acids in piglet formulas (OAF, oleic acid-based formula; PAF, palmitic acid-based formula; MCFAF, medium chain fatty acid-based formula; LAF, linoleic acid-based formula).

Based on their fatty acid composition, commercial piglet formulas were classified into four groups (R = 0.826, *p* < 0.001; Figure 1): oleic acid-based formula (OAF, *n* = 9), palmitic acid-based formula (PAF, *n* = 5), medium chain fatty acid formula-based formula (MCFAF, *n* = 4), and linoleic acid-based formula (LAF, *n* = 4). High oleic oils, palm oil and its fractions, coconut oil or palm kernel oil, and linoleic acid-rich oils might be the main fat ingredients in the manufacture of these respective formula types.

### 3.2. Fatty Acids in Sow Colostrum, Sow Milk and Piglet Formulas

#### 3.2.1. Saturated Fatty Acids

Saturated fatty acid (SFA) composition of sow colostrum, sow milk, and commercial piglet formulas are presented in Table 1. The levels of SFAs in all piglet formulas, except for LAF products, were significantly greater than those in sow milk and sow colostrum fat. Furthermore, fat from both formulas and sow milk contained low concentrations of short chain (<8 carbons) saturated fatty acids (SC-SFAs), i.e., <1% of total SFA. Medium chain (8–14 carbons) saturated fatty acids (MC-SFAs, mainly C8:0, C10:0, C12:0, and C14:0 in the present study) were the notable SFA species in all studied samples. The levels of MC-SFA’s in sow milk fat (2.4–3.1%) were significantly lower than those in formulas (7.9–27.2%). Long chain (>14 carbons) saturated fatty acids (LC-SFAs), mainly C16:0 and C18:0, were the most abundant SFA species in the fat of sow colostrum and sow milk, reaching 33.3% and 35.7%, respectively. The SFA composition of piglet formula samples was quite different, depending on the included fat ingredients. For instance, PAF products contained a significantly higher level (46.1%) of SFA than sow milks, while the concentration in LAF products was lower, i.e., 26.1%. In contrast, the SFA levels in OAF and MCFAF products were closer to sow colostrum and sow milk.

#### 3.2.2. Unsaturated Fatty Acids

A total of 15 UFAs were detected in the fat from sow colostrum, sow milk and piglet formulas, and their concentrations are presented in Table 2. Sow colostrum and milk fat contained higher UFA levels (64.3–66.6%) than all the studied formulas (40.5–53.3%) except for the LAF formula products. The significant differences were mainly ascribed to C16:1, C18:1, and C18:2. C16:1 was one of the most important monounsaturated fatty acids in sow milk, with concentrations of 3.6% in sow colostrum fat and 7.4% in sow milk fat. In contrast, less than 1% of C16:1 was detected in the fat of piglet formulas. C18:1 was generally the most abundant fatty acid, ranging from 27.0 to 35.2% of total fat in formulas; while its levels increased from 28.6% in sow colostrum fat to 37.4% in sow milk fat. Sow colostrum fat showed significantly higher concentrations of polyunsaturated fatty acids (PUFAs; 33.3%) than the fat of all piglet formulas (7.3–17.0%), except for LAF formulas, whereas the PUFA concentration was also significantly lower in sow milk fat (18.4%) than in sow colostrum fat samples. C18:2 was the predominant species in PUFAs, reaching 27.5% of total fat in colostrum and 15.3% in milk. It is also suggested that linolenic acid-rich oils and lipids from marine organism should be included in formulas for newborn piglets as supplements, to increase C18:3, C20:4, C20:5 and C22:6, making their concentrations close to colostrum.

#### 3.2.3. Principal Component Analysis of Fatty Acids in Sow Colostrum and Milk and Piglet Formulas

**Figure 2:**
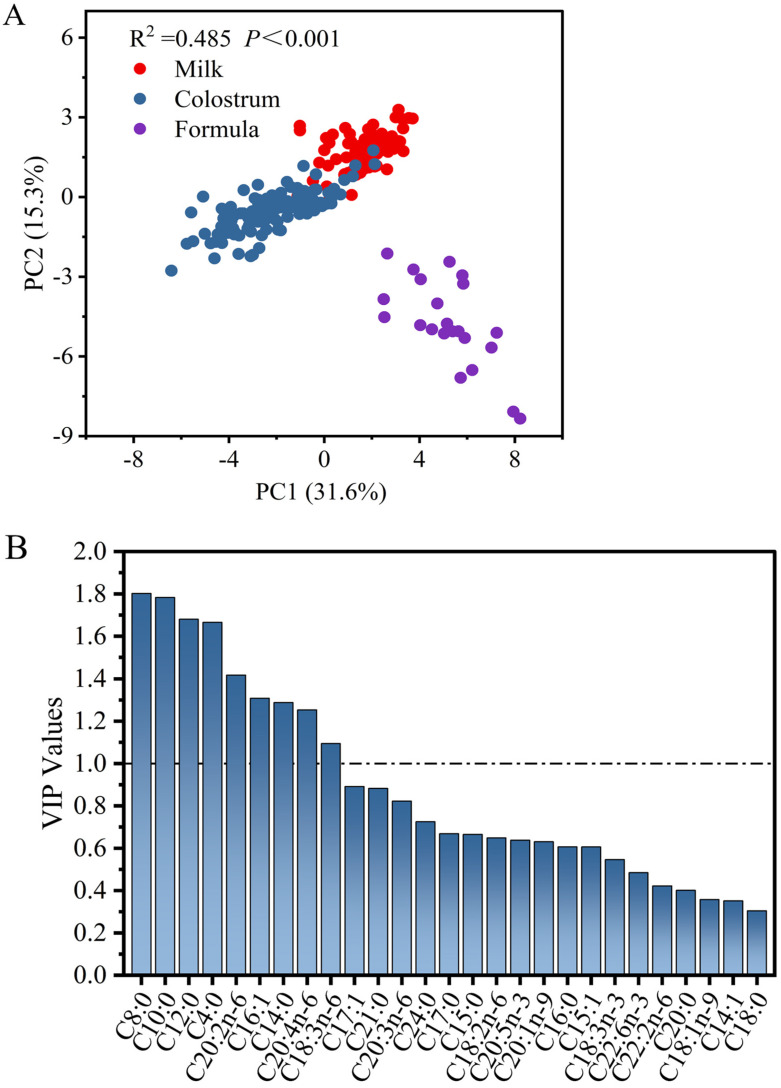
Principal component analysis (PCA) (**A**) and variable importance in the projection (VIP) values (**B**) of fatty acid compositions in sow colostrum, sow milk and piglet formulas.

PCA of fatty acids of sow milks and formulas are shown in Figure 2A. Three distinct groups, i.e., sow colostrum, sow milk, and piglet formulas, were found based on their fatty acid profiles (R^2^ = 0.485, *p* < 0.001). The screening result of differential markers of fatty acids between sow milks and formulas are further presented in Figure 2B. There are 9 kinds of differentiating fatty acids (VIP > 1) between sow milks and piglet formulas, including C8:0, C10:0, C12:0, C4:0, C20:2, C16:1, C14:0, C20:4, and C18:3; of which, the former four exhibited larger VIP values, i.e., more than 1.5. Higher levels of MC-SFAs in formulas were responsible for the difference. In contrast, PUFAs, especially C18:3 and C20:4, were the limitations in current piglet formulas.

### 3.3. sn-2 Fatty Acids of Sow Colostrum and Milk and Piglet Formulas

#### 3.3.1. *sn*-2 Saturated Fatty Acids

SFAs located at the *sn*-2 position in piglet formulas, sow milk, and colostrum are shown in Table 3. Although the overall SFA content at the *sn*-2 position in piglet formulas (45.3–68.6%), except LAF formulas, were close to that in sow colostrum (50.8%) and sow milk (57.3%), the fatty acid compositions were quite different, mainly in terms of MC-SFAs and LC-SFAs. Both sow colostrum and sow milk fat showed higher concentrations of LC-SFAs at the sn-2 position (47.5 and 52.7%, respectively) compared with the four formula types (24.5–39.3%). The most abundant sn-2 LC-SFAs in sow milk was C16:0 (43.0–48.4%), which was significantly higher than in piglet formulas (20.6–30.9%); in contrast, piglet formulas, including OAF, PAF, and MCFAF, contained higher levels of sn-2 C18:0 (5.6–12.8%) than sow milks (3.4–3.6%). In contrast to LC-SFAs, the sn-2 levels of MC-SFAs in sow colostrum and milk (3.3–4.6%) were significantly lower than those in piglet formulas (11.4–29.3%). C12:0, coming from coconut oils or palm kernel oils, was the typical sn-2 MC-SFA in formulas (6.9–20.3%), whereas the main sn-2 MC-SFAs in sow colostrum and milk was C14:0 (3.1–4.2%).

#### 3.3.2. *sn*-2 Unsaturated Fatty Acids

The distribution of UFAs at the *sn*-2 position in sow colostrum, milk, and piglet formulas are presented in Table 4. Percentages of *sn*-2 MUFAs (36.6–42.3%) in all the formulas, except MCFAF formulas, were significantly greater than those in sow colostrum and milk (22.7–26.5%). C18:1, the most abundant MUFA, was the main determinant for these differences. Similar to the total fatty acid compositions, 3.7–5.3% of C16:1 was detected at the *sn*-2 position in sow colostrum and milk, whereas <1% sn-2 C16:1 was found in the piglet formulas. Percentages of PUFAs at the *sn*-2 position comprised 24.2% in sow colostrum and 14.5% in sow milk, which were significantly higher than those in piglet formulas (6.2–9.1%) except for LAF. The differences were in accordance with the changes of *sn*-2 C18:2.

#### 3.3.3. Principal Component Analysis of *sn*-2 Fatty Acids in Sow Colostrum and Milk and Piglet Formulas

**Figure 3:**
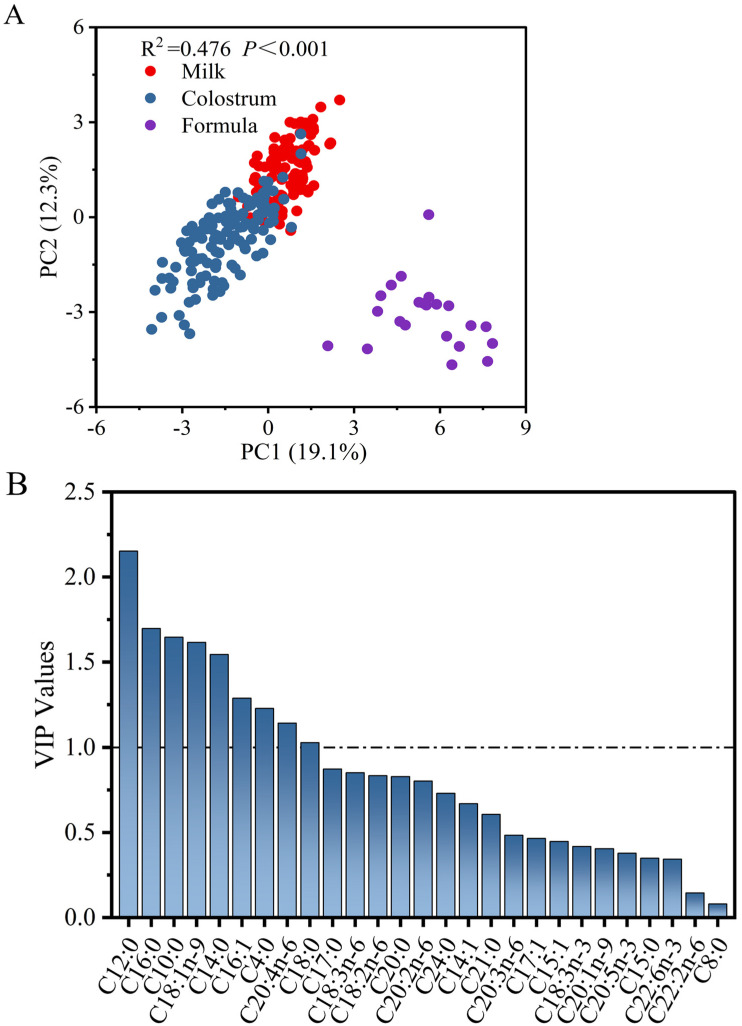
Principal component analysis (PCA) (**A**) and variable importance in the projection (VIP) values (**B**) of *sn*-2 fatty acid composition in sow colostrum, sow milk and piglet formulas.

Sow colostrum, sow milk, and piglet formulas were partitioned based on their *sn*-2 fatty acids (R^2^ = 0.476, *p* < 0.001) as shown in Figure 3A, indicating remarkable differences in the distribution of fatty acids between sow milks and formulas. Differential markers of *sn*-2 fatty acids between sow milk, colostrum, and formulas are presented in Figure 3B. There were nine major kinds of differential *sn*-2 fatty acids (VIP values > 1) between sow colostrum, sow milk, and piglet formulas. The differentiating markers with higher VIP values were C12:0, C16:0, C10:0, C18:1, and C14:0. In this case, higher levels of *sn*-2 C16:0 and *sn*-2 C14:0 in sow milks, and higher levels of *sn*-2 C18:1 in formulas, contributed to their main differences.

#### 3.3.4. Relative Percentage of Fatty Acids at the sn-2 Position

The relative percentage of each fatty acid distributed at the *sn*-2 position was computed as 100 × (M/3)/N, where M is % *sn*-2 fatty acid (Table 3 and Table 4), and N is % total fatty acid (Table 1 and Table 2). The calculated results are important to better understand the stereochemical structures of TAGs, which are presented in Table 5. C16:0, the most abundant SFA, was predominantly located at the *sn*-2 position of TAG molecules in both sow colostrum and milk (63.2–63.4%), whereas the most abundant UFAs in sow milk fats, C18:1 and C18:2, were mainly found at the *sn*-1,3 positions.

**Figure 4:**
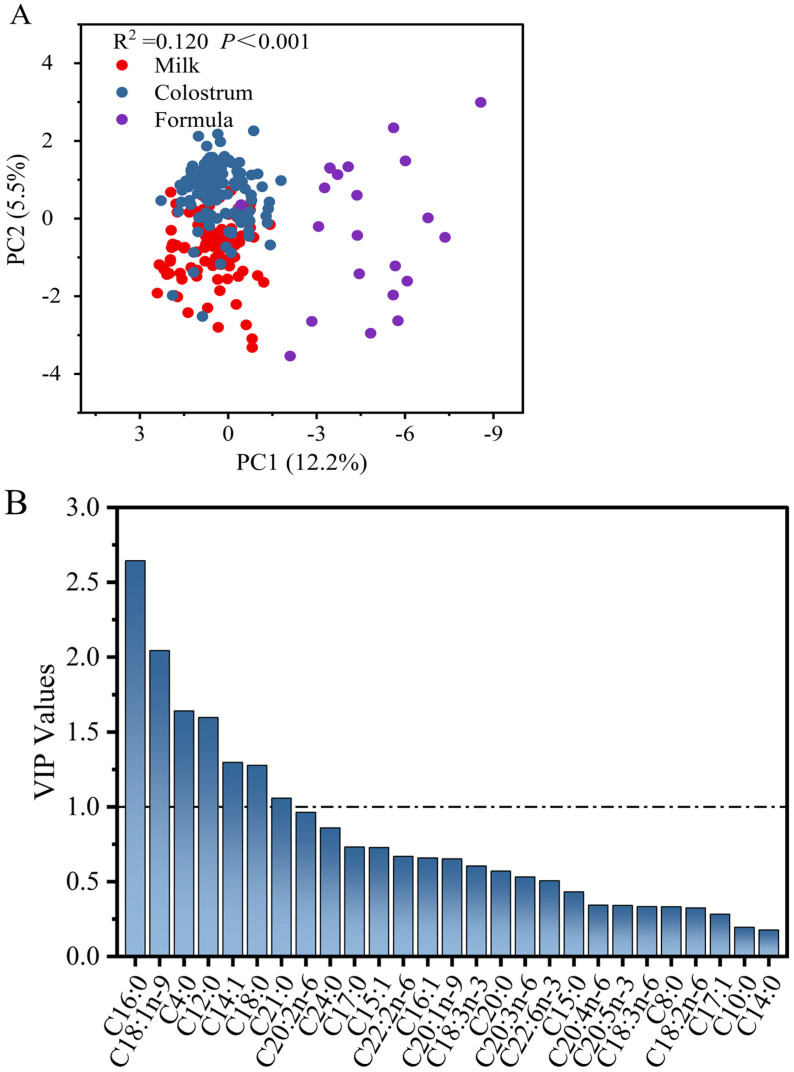
Principal component analysis (**A**) and variable importance in the projection (VIP) values (**B**) of relative percentage of each fatty acid at the sn-2 position in sow colostrum, sow milk, and piglet formulas.

Figure 4 depicts differential indicators of the relative percentage of each fatty acid at the sn-2 location in sow milk and formulas. Three distinct groups were also found based on distributions of fatty acids (R^2^ = 0.120, *p* < 0.001), which were accordance with the data from fatty acids and *sn*-2 fatty acids mention above. C16:0 and C18:1 were confirmed as the major differential fatty acids (VIP > 1), as expected, in Figure 4B.

### 3.4. Triacylglycerols in Sow Colostrum, Sow Milk and Piglet Formulas

The major TAG species and their contents (>1%) in fats from sow milks and piglet formulas are presented in Table 6. There were obvious differences in TAGs containing palmitic acid between formulas and sow milks. TAGs containing SC-SFAs and MC-SFAs from coconut oil or palm kernel oil were abundant in formula fats, e.g., LaLaCy, CaCaM, LaLaCa, LaLaLa, and LaLaM, etc. Together, these TAGs accounted for 6.1%, 7.0%, 24.2%, and 4.9% of total TAGs in OAF, PAF, MCFAF, and LAF formulas, respectively. However, these TAGs were scarcely determined in sow milk fats; whereas LC-SFAs were the abundant fatty acid species attached to TAG molecules of sow milk fats. In particular, both colostrum and milk fats contained significantly higher amounts of TAGs, such as OPL, OPO, LPL, and OPPo, ranging from 37.8 to 38.0%, Most of these molecules were characterized as palmitic acid connected to the sn-2 position and unsaturated fatty acids located at the sn-1,3 positions. The structural isomer was calculated following the abundance ratio of fragments produced by the loss of different positions (sn-1, sn-2, and sn-3), and also agreed with sn-2 fatty acid compositions of sow milk fats and formula fats [12,13,15,30]. TAGs consisting of palmitic acid, one saturated fatty acid, and one unsaturated fatty acid are another important component, e.g., OPP, PPL, SPO, and OPM, accounting for 19.0% and 31.4% in colostrum and milk, respectively. The levels of these TAGs in piglet formula fats varied from 16.2 to 41.3%, depending on the used fats and oils. In addition, piglet formula fats contained higher amounts (3.4–11.9%) of TAGs with palmitic acid and two saturated fatty acids (e.g., PLaLa, PMLa, and PPS) than sow milk fats (1.2–1.9%).

**Figure 5:**
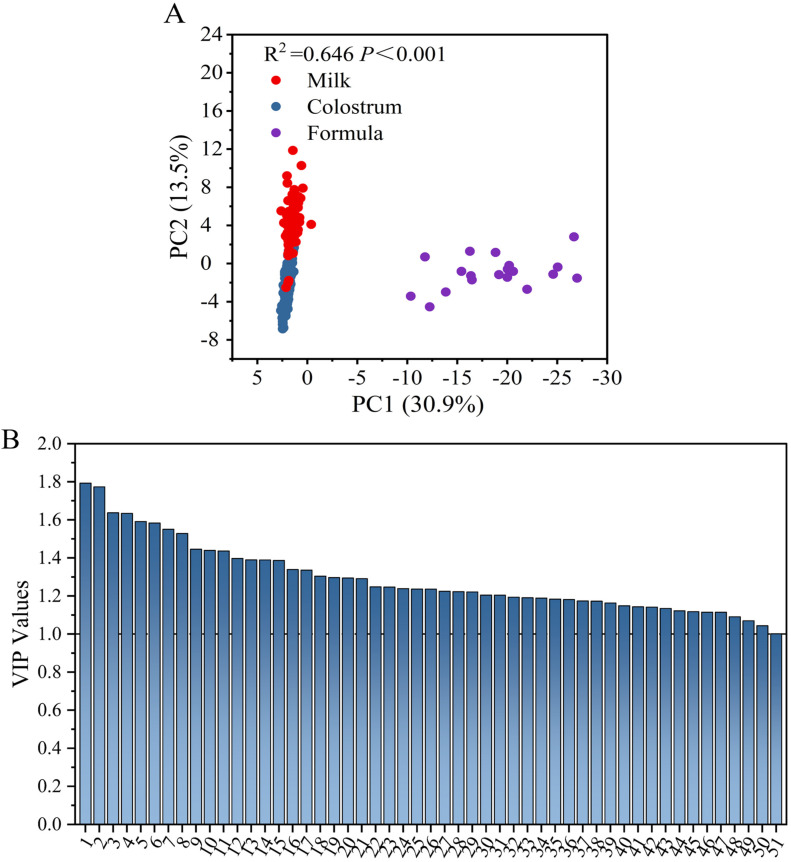
Principal component analysis (PCA) (**A**) and variable importance in the projection (VIP) values (**B**) of triacylglycerols in sow colostrum, sow milk, and piglet formulas. The structural isomer of TAG species was identified by UPLC-Q-TOF-MS. 1, LOP; 2, OOP; 3, OPL; 4, OLaP; 5, SOO; 6, PPBu; 7, LLP; 8, OPoO; 9, EPO; 10, SLaLa; 11, SOS; 12, OMBu; 13, PBuM; 14, LaLaLa; 15, OCoP; 16, SPL; 17, OPO; 18, OOBu; 19, PLaLa; 20, OBuP; 21, LaLaM; 22, MMP; 23, PPLa; 24, EOO; 25, OCyP; 26, SSP; 27, OLaBu; 28, OMCo; 29, LPPo; 30, OML; 31, PPCo; 32, LPBu; 33, OPPo; 34, OBuS; 35, OLaM; 36, SLO; 37, SCoP; 38, OMO; 39, CaCaM; 40, LaLaCo; 41, MMLa; 42, MMM; 43, OODo; 44, PMLa; 45, OSL; 46, LaLaCa; 47, LaLaCy; 48, MMCo; 49, PCoLa; 50, LLnLn; 51, PPCa. Bu, C4:0; Co, C6:0; Cy, C8:0; Ca, C10:0; La, C12:0; M, C14:0; P, C16:0; Po, C16:1; S, C18:0; L, C18:1; O, C18:2; Ln, C18:3; E, C20:0; Do, C22:0.

Colostrum, milk, and formulas were also significantly distinguished based on their TAG compositions, which are presented in Figure 5A. TAG species with VIP values > 1 were regarded as potential markers between formulas and sow milks (Figure 5B). There were 51 differential TAG species that showed significant differences between formulas and sow milks, among which OPL and OPO were the most important differential TAGs. The quantity was far more than the indices discussed above, such as fatty acids and sn-2 fatty acids. It was also concluded from previous report that the distribution of fatty acids on TAG molecules were significantly different between sow milks and formulas [12].

## 4. Discussion

Piglet formulas were quite different depending on their included fat ingredients (Figure 1). High oleic oils, palm oil and its fractions, coconut oil or palm kernel oil, and linoleic acid-rich oils might be the main fat ingredients in the manufacture of commercial formulas.

The total fatty acid compositions showed great differences between sow milks and piglet formulas (Table 1 and Table 2). Table 1 shows that the levels of MC-SFAs in sow milk fat were significantly lower than those in formulas. These are important findings because it is well-known that MC-SFAs are absorbed more readily compared to LC-SFAs, providing necessary energy in time for newborns [33]. However, high content of MCFA increases the osmolality of the preparation, since it is associated with higher risk of osmotic diarrhea, which should also be taken into account during commercial formula production [33]. For unsaturated fatty acids, it was found that C16:1 was one of the main monounsaturated fatty acids in sow milks (Table 2). However, less than 1% of C16:1 was detected in the piglet formulas (Table 2). C16:1 has recently received increasing interest as a promising anti-inflammatory lipid that may help to ameliorate metabolic disorders, which is often ignored in developing piglet formulas [32,34].

In addition, there was a significant difference in the content of FAs in sow milk from sows in China studied here, compared to those in other regions. C14:0 and C16:0 levels in sow milk from Denmark [35] (6.7% and 30.1%) were higher than those from China of our study (2.7% and 25.6%; Table 1), whereas the C16:1 level in sow milk from our study (7.4%; Table 2) was higher than that from Poland [36] (3.9%). The C18:2 contents in sow milk fat from Canada [37] (8.2%) and Poland [36] (7.4%) were lower than those from China in our study (15.3%; Table 2). Therefore, regional dietary and sow milk fat characteristics should be considered when developing commercial formulas of piglets for specific geographies, e.g., China.

In this study, higher levels of sn-2 C16:0 and sn-2 C14:0 in sow milks, and higher levels of sn-2 C18:1 in formulas, contributed to their main differences (Figure 3A). Table 3 showed the most abundant sn-2 LC-SFA in sow milk was C16:0, which was significantly higher than those in formulas; C16:0 was predominantly located at the sn-2 positions of TAG molecules in both sow colostrum and milk, whereas C18:1 and C18:2 were mainly found at the sn-1,3 positions (Table 3 and Table 4). The result was consistent with previous reports, who also pointed that C16:0 was mainly located at the sn-2 position in sow milks, whereas they were more likely linked to the sn-1,3 position in formulas [12,13,14]. Such a unique distribution contributed to increasing the absorption of fatty acids and calcium by infants, as well as improving their stool consistency [20]. The difference in fatty acid distribution of fats in sow milks and formulas, especially sn-O/P/O and sn-O/O/P, resulted in some of the major differential pathways in piglets, mainly digestion and absorption [38,39]. Lower amounts of such TAGs were presented in formula fats, indicating common vegetable oils were their main fat sources [40].

## 5. Conclusions

Three distinct groups, mainly colostrum, milk, and formulas, were classified according to their fatty acid compositions and distributions, as well as TAG species. Compared with sow milk fats, formula fats contained significantly higher levels of SC-SFAs and MC-SFAs, while their UFA proportions were significantly lower. Although palmitic acid, oleic acid, and linoleic acid were the dominant fatty acids in all the fats from formulas and sow milks, nearly 65% of palmitic acid was distributed at the sn-2 position of TAGs in sow milk fats, which was quite different from that in formula fats (less than 40%). UPLC-Q-TOF-MS further confirmed that stereochemical structure of TAGs, e.g., OPL and OPO, were the differential markers, which could distinguish sow milk fats from commercial oils used in formulas. It is suggested to pay more attention to TAG species in designing oils for piglet formulas.

## Figures and Tables

**Table 1:** Saturated fatty acids in piglet formulas, sow colostrum, and sow milk.

Fatty Acids	OAF	PAF	MCFAF	LAF	Colostrum	Milk
C4:0	0.7 ± 0.3 ^ab^	0.3 ± 0.3	0.2 ± 0.2	0.3 ± 0.3	ND	ND
C8:0	0.7 ± 0.2 ^ab^	0.8 ± 0.4 ^ab^	1.6 ± 0.4 ^ab^	0.6 ± 0.3	0.1 ± 0.1	0.0 ± 0.0
C10:0	1.1 ± 0.3 ^ab^	1.1 ± 0.6 ^ab^	1.7 ± 0.4 ^ab^	0.7 ± 0.2 ^ab^	0.1 ± 0.2	0.1 ± 0.1
C12:0	4.8 ± 2.2 ^ab^	6.0 ± 3.4 ^ab^	16.0 ± 2.7 ^ab^	3.9 ± 2.0	0.3 ± 0.3	0.3 ± 0.2
C14:0	3.9 ± 0.9 ^ab^	4.7 ± 1.6 ^a^	7.9 ± 1.6 ^ab^	2.7 ± 0.7 ^a^	2.0 ± 0.5	2.7 ± 0.5
C15:0	0.2 ± 0.1 ^a^	0.2 ± 0.2 ^a^	0.1 ± 0.1 ^ab^	0.1 ± 0.1 ^ab^	0.4 ± 0.2	0.3 ± 0.1
C16:0	26.4 ± 2.7 ^a^	38.1 ± 5.2 ^ab^	22.3 ± 2.4 ^b^	20.6 ± 1.8 ^b^	22.7 ± 3.3	25.6 ± 2.5
C17:0	0.2 ± 0.1 ^ab^	0.2 ± 0.1 ^ab^	0.2 ± 01 ^ab^	0.2 ± 0.0 ^ab^	0.5 ± 0.3	0.3 ± 0.1
C18:0	6.2 ± 1.6	7.3 ± 1.6 ^b^	7.9 ± 1.9 ^ab^	4.8 ± 0.9 ^a^	6.0 ± 1.0	5.8 ± 1.1
C20:0	0.3 ± 0.0 ^b^	0.3 ± 0.1 ^b^	0.3 ± 0.0	0.3 ± 0.0 ^b^	0.3 ± 0.3	0.2 ± 0.1
C21:0	0.0 ± 0.0 ^ab^	0.0 ± 0.0 ^ab^	0.0 ± 0.0 ^ab^	0.0 ± 0.0 ^ab^	0.5 ± 0.2	0.2 ± 0.1
C24:0	0.1 ± 0.1 ^a^	0.1 ± 0.0 ^a^	0.1 ± 0.1 ^a^	0.1 ± 0.1 ^a^	0.4 ± 0.2	0.1 ± 0.1
SFAs	44.5 ± 3.1 ^ab^	58.9 ± 6.8 ^ab^	58.1 ± 4.7 ^ab^	34.2 ± 2.8	33.3 ± 3.7	35.7 ± 2.7
SC-SFAs	0.7 ± 0.3 ^ab^	0.3 ± 0.3	0.2 ± 0.2	0.3 ± 0.3	ND	ND
MC-SFAs	10.4 ± 3.0 ^ab^	12.5 ± 5.2 ^ab^	27.2 ± 5.0 ^ab^	7.9 ± 3.2	2.4 ± 0.8	3.1 ± 0.7
LC-SFAs	33.4 ± 3.8 ^a^	46.1 ± 6.2 ^ab^	30.8 ± 3.5	26.1 ± 2.4 ^ab^	30.8 ± 3.6	32.5 ± 2.4

ND: Not detected. Data are given as weight %. Values are average values ± standard deviation (Mean ± SD). a and b mean that significant differences (*p* < 0.05) were detected between formulas and colostrum, and formulas and milk, respectively. Abbreviations: OAF, oleic acid-based formula; PAF, palmitic acid-based formula; MCFAF, medium chain fatty acid formula-based formula; LAF, linoleic acid-based formula; SFAs, saturated fatty acids; SC-SFAs, short chain (<8 carbons)-SFAs; MC-SFAs, medium chain (8–14 carbons)-SFAs; LC-SFAs, long chain (>14 carbons)-SFAs.

**Table 2:** Unsaturated fatty acids in piglet formulas, sow colostrum, and sow milk.

Fatty Acids	OAF	PAF	MCFAF	LAF	Colostrum	Milk
C14:1	0.1 ± 0.1 ^b^	0.1 ± 0.1	0.1 ± 0.1	0.1 ± 0.0 ^b^	0.1 ± 0.1	0.3 ± 0.1
C15:1	0.0 ± 0.0 ^a^	0.0 ± 0.1	ND	0.0 ± 0.0 ^a^	0.2 ± 0.2	0.1 ± 0.1
C16:1	0.7 ± 0.2 ^ab^	0.4 ± 0.4 ^ab^	0.4 ± 0.2 ^ab^	0.4 ± 0.1 ^ab^	3.6 ± 0.9	7.4 ± 1.8
C17:1	0.1 ± 0.0 ^ab^	0.0 ± 0.0 ^ab^	0.0 ± 0.0 ^ab^	0.0 ± 0.0 ^ab^	0.4 ± 0.3	0.4 ± 0.3
C18:1	35.2 ± 5.4 ^a^	32.5 ± 5.7	27.0 ± 2.5 ^b^	31.6 ± 5.3	28.6 ± 4.4	37.4 ± 6.5
C18:2	14.9 ± 2.6 ^a^	6.6 ± 1.5 ^ab^	12.5 ± 1.8 ^a^	28.3 ± 4.2 ^b^	27.5 ± 5.0	15.3 ± 5.6
C18:3 n6	0.0 ± 0.0 ^ab^	0.0 ± 0.0 ^ab^	0.0 ± 0.0 ^ab^	0.0 ± 0.0 ^ab^	0.4 ± 0.2	0.4 ± 0.2
C18:3 n3	1.6 ± 0.2 ^ab^	0.6 ± 0.5 ^a^	1.3 ± 0.4 ^a^	4.1 ± 1.1 ^ab^	2.5 ± 1.6	0.9 ± 0.6
C20:1	0.2 ± 0.1 ^ab^	0.1 ± 0.1 ^ab^	0.2 ± 0.1 ^ab^	0.2 ± 0.1 ^ab^	0.4 ± 0.2	0.4 ± 0.3
C20:2	0.0 ± 0.0 ^ab^	0.0 ± 0.0 ^ab^	0.0 ± 0.0 ^ab^	0.0 ± 0.0 ^ab^	0.8 ± 0.2	0.5 ± 0.1
C20:3	0.0 ± 0.0 ^ab^	0.0 ± 0.0 ^ab^	ND	0.0 ± 0.0 ^ab^	0.3 ± 0.2	0.1 ± 0.1
C20:4	0.2 ± 0.1 ^ab^	0.0 ± 0.0 ^ab^	0.1 ± 0.1 ^ab^	0.1 ± 0.1 ^ab^	1.3 ± 0.4	1.0 ± 0.4
C20:5	0.1 ± 0.0 ^a^	0.1 ± 0.1 ^a^	0.0 ± 0.0 ^a^	0.2 ± 0.1	0.2 ± 0.1	0.1 ± 0.1
C22:2	0.0 ± 0.0	0.0 ± 0.0	0.1 ± 0.1	0.1 ± 0.0	0.1 ± 0.1	0.0 ± 0.0
C22:6	0.1 ± 0.1	0.0 ± 0.0 ^ab^	0.1 ± 0.1	0.0 ± 0.0 ^ab^	0.2 ± 0.1	0.1 ± 0.1
UFAs	53.3 ± 3.3 ^ab^	40.5 ± 6.0 ^ab^	41.8 ± 3.2 ^ab^	65.1 ± 3.0	66.6 ± 4.0	64.3 ± 2.8
MUFAs	36.3 ± 5.2 ^b^	33.2 ± 5.7 ^b^	27.7 ± 2.8 ^ab^	32.3 ± 5.2 ^b^	33.3 ± 4.6	45.9 ± 6.0
PUFAs	17.0 ± 2.6 ^a^	7.3 ± 1.4 ^ab^	14.1 ± 2.1 ^ab^	32.8 ± 5.2 ^b^	33.3 ± 6.0	18.4 ± 6.1

ND: Not detected. Data are given as weight %. Values are average values ± standard deviation (Mean ± SD). a and b mean that significant differences (*p* < 0.05) were detected between formulas and colostrum, and formulas and milk, respectively. Abbreviations: OAF, oleic acid-based formula; PAF, palmitic acid-based formula; MCFAF, medium chain fatty acid formula-based formula; LAF, linoleic acid-based formula; UFAs, unsaturated fatty acids; MUFAs, monounsaturated fatty acids; PUFAs, polyunsaturated fatty acids.

**Table 3:** Saturated fatty acids distributed at the sn-2 position in piglet formulas, sow colostrum, and sow milk.

Fatty Acids	OAF	PAF	MCFAF	LAF	Colostrum	Milk
C4:0	0.2 ± 0.3 ^ab^	ND	0.1 ± 0.1 ^ab^	0.1 ± 0.1 ^ab^	ND	ND
C8:0	0.0 ± 0.0	0.1 ± 0.1	0.1 ± 0.1	0.0 ± 0.0	0.0 ± 0.0	0.1 ± 0.8
C10:0	0.4 ± 0.2 ^ab^	0.4 ± 0.4	0.3 ± 0.2	0.2 ± 0.1 ^ab^	0.0 ± 0.1	0.0 ± 0.1
C12:0	8.1 ± 2.9 ^ab^	7.8 ± 5.4 ^ab^	20.3 ± 2.6 ^ab^	6.9 ± 3.6 ^ab^	0.2 ± 0.1	0.3 ± 0.1
C14:0	7.2 ± 1.9 ^ab^	5.5 ± 2.4	8.7 ± 1.9 ^ab^	4.2 ± 1.4	3.1 ± 0.7	4.2 ± 0.7
C15:0	0.3 ± 0.1	0.2 ± 0.2	0.2 ± 0.1 ^a^	0.2 ± 0.1 ^a^	0.3 ± 0.1	0.2 ± 0.1
C16:0	31.0 ± 11.7 ^ab^	23.8 ± 3.5 ^ab^	25.9 ± 5.7 ^ab^	20.6 ± 8.7 ^ab^	43.0 ± 5.3	48.4 ± 5.4
C17:0	0.1 ± 0.1 ^ab^	0.2 ± 0.1 ^a^	0.2 ± 0.1 ^a^	0.2 ± 0.1 ^ab^	0.3 ± 0.1	0.3 ± 0.1
C18:0	5.6 ± 4.0	7.0 ± 2.3 ^ab^	12.8 ± 4.9 ^ab^	3.4 ± 1.3	3.4 ± 1.6	3.6 ± 1.8
C20:0	0.2 ± 0.1 ^ab^	0.4 ± 0.4	0.2 ± 0.2	0.2 ± 0.1 ^ab^	0.1 ± 0.1	0.1 ± 0.1
C21:0	0.0 ± 0.0	0.0 ± 0.0	0.0 ± 0.0	0.0 ± 0.0	0.1 ± 0.2	0.1 ± 0.1
C24:0	0.0 ± 0.0 ^ab^	0.0 ± 0.0 ^ab^	0.0 ± 0.0 ^ab^	ND	0.3 ± 0.2	0.1 ± 0.1
SFAs	52.9 ± 14.0	45.3 ± 8.6 ^ab^	68.6 ± 7.7 ^ab^	36.0 ± 10.3 ^ab^	50.8 ± 5.8	57.3 ± 6.6
SC-SFAs	0.2 ± 0.3 ^ab^	ND	0.1 ± 0.1	0.1 ± 0.1	ND	ND
MC-SFAs	15.6 ± 3.0 ^ab^	13.8 ± 6.4 ^ab^	29.3 ± 1.8 ^ab^	11.4 ± 4.1 ^ab^	3.3 ± 0.8	4.6 ± 1.0
LC-SFAs	37.1 ± 15.0 ^b^	31.5 ± 4.9 ^ab^	39.3 ± 9.1 ^b^	24.5 ± 9.4 ^ab^	47.5 ± 5.7	52.7 ± 6.4

ND: Not detected. Data are given as weight %. Values are average values ± standard deviation (Mean ± SD). a and b mean that significant differences (*p* < 0.05) were detected between formulas and colostrum, and formulas and milk, respectively. Abbreviations: OAF, oleic acid-based formula; PAF, palmitic acid-based formula; MCFAF, medium chain fatty acid formula-based formula; LAF, linoleic acid-based formula; SFAs, saturated fatty acids; SC-SFAs, short chain (<8 carbons)-SFAs; MC-SFAs, medium chain (8–14 carbons)-SFAs; LC-SFAs, long chain (>14 carbons)-SFAs.

**Table 4:** Unsaturated fatty acids distributed at the sn-2 position in piglet formulas, sow colostrum, and sow milk.

Fatty Acids	OAF	PAF	MCFAF	LAF	Colostrum	Milk
C14:1	0.1 ± 0.1	0.2 ± 0.2 ^a^	0.1 ± 0.1	0.1 ± 0.1	0.0 ± 0.0	0.1 ± 0.1
C15:1	ND	ND	ND	ND	0.0 ± 0.0	0.0 ± 0.0
C16:1	0.6 ± 0.2 ^ab^	0.5 ± 0.4 ^ab^	0.8 ± 0.5 ^ab^	0.5 ± 0.2 ^ab^	3.7 ± 1.0	6.3 ± 2.6
C17:1	0.0 ± 0.0 ^b^	0.0 ± 0.1 ^b^	0.0 ± 0.0 ^b^	0.0 ± 0.0 ^b^	0.3 ± 0.5	0.2 ± 0.2
C18:1	35.8 ± 14.3 ^ab^	41.5 ± 7.4 ^ab^	21.7 ± 4.7	36.1 ± 3.2 ^ab^	18.6 ± 3.8	19.7 ± 4.8
C18:2	7.0 ± 3.3 ^ab^	8.4 ± 3.1 ^a^	5.7 ± 1.6 ^ab^	23.6 ± 6.5 ^b^	21.2 ± 4.5	12.7 ± 5.2
C18:3n6	0.0 ± 0.0 ^ab^	0.0 ± 0.0 ^ab^	0.0 ± 0.0 ^ab^	0.0 ± 0.0 ^ab^	0.3 ± 0.2	0.2 ± 0.3
C18:3n3	0.4 ± 0.3 ^a^	0.5 ± 0.8	0.4 ± 0.1 ^a^	1.9 ± 1.0 ^b^	1.1 ± 0.9	0.4 ± 0.4
C20:1	0.1 ± 0.0	0.1 ± 0.0	0.0 ± 0.0	0.1 ± 0.0	0.1 ± 0.1	0.1 ± 0.1
C20:2	0.0 ± 0.0 ^ab^	0.1 ± 0.1 ^a^	0.0 ± 0.0 ^ab^	0.1 ± 0.0 ^ab^	0.3 ± 0.2	0.1 ± 0.1
C20:3	0.0 ± 0.0 ^a^	ND	0.0 ± 0.0 ^a^	0.0 ± 0.0 ^a^	0.1 ± 0.1	0.0 ± 0.0
C20:4	0.1 ± 0.1 ^ab^	0.0 ± 0.0 ^ab^	0.0 ± 0.0 ^ab^	0.1 ± 0.0 ^ab^	1.0 ± 0.5	0.9 ± 0.6
C20:5	0.1 ± 0.1	0.0 ± 0.0	0.0 ± 0.0	0.0 ± 0.0	0.1 ± 0.1	0.1 ± 0.0
C22:2	0.0 ± 0.0	0.0 ± 0.0	0.0 ± 0.0	0.0 ± 0.0	0.0 ± 0.0	0.0 ± 0.1
C22:6	0.1 ± 0.1	0.0 ± 0.0 ^ab^	0.0 ± 0.0 ^ab^	0.0 ± 0.0 ^ab^	0.1 ± 0.1	0.1 ± 0.1
UFAs	44.4 ± 15.0	51.3 ± 3.6 ^ab^	28.8 ± 6.7 ^ab^	62.5 ± 3.6 ^ab^	46.9 ± 5.7	40.9 ± 6.6
MUFAs	36.6 ± 4.2 ^ab^	42.3 ± 7.5 ^ab^	22.6 ± 5.2	36.8 ± 3.2 ^ab^	22.7 ± 4.3	26.5 ± 5.4
PUFAs	7.7 ± 3.5 ^ab^	9.1 ± 3.8 ^ab^	6.2 ± 1.8 ^ab^	25.7 ± 8.6 ^b^	24.2 ± 5.2	14.5 ± 4.9

ND: Not detected. Data are given as weight %. Values are average values ± standard deviation (Mean ± SD). a and b mean that significant differences (*p* < 0.05) were detected between formulas and colostrum, and formulas and milk, respectively. Abbreviations: OAF, oleic acid-based formula; PAF, palmitic acid-based formula; MCFAF, medium chain fatty acid formula-based formula; LAF, linoleic acid-based formula; UFAs, unsaturated fatty acids; MUFAs, monounsaturated fatty acids; PUFAs, polyunsaturated fatty acids.

**Table 5:** Relative percentages of each fatty acids at the *sn*-2 position in piglet formulas, sow colostrum and sow milk.

Fatty Acid	OAF	PAF	MCFAF	LAF	Colostrum	Milk
C4:0	13.3 ± 15.8 ^ab^	ND	4.2 ± 7.4	10.8 ± 18.6	ND	ND
C8:0	0.4 ± 0.8 ^ab^	3.8 ± 3.4	1.2 ± 2.0	2.1 ± 3.6	3.6 ± 10.1	4.6 ± 11.4
C10:0	12.0 ± 4.5	13.8 ± 7.3	6.1 ± 4.3	9.5 ± 4.8	6.5 ± 16.9	10.4 ± 12.0
C12:0	58.2 ± 10.3 ^ab^	47.5 ± 19.9 ^ab^	42.7 ± 2.5 ^ab^	60.6 ± 9.6 ^ab^	24.2 ± 13.7	25.1 ± 17.8
C14:0	63.0 ± 15.3	39.8 ± 10.2 ^ab^	38.8 ± 13.4	52.7 ± 17.4	53.1 ± 8.7	52.0 ± 7.7
C15:0	41.2 ± 18.6 ^ab^	13.1 ± 16.4 ^ab^	25.6 ± 25.6	33.5 ± 19.8	30.6 ± 14.1	26.1 ± 12.6
C16:0	38.5 ± 12.4 ^ab^	21.1 ± 4.0 ^ab^	39.1 ± 9.9 ^ab^	34.3 ± 16.7 ^ab^	63.4 ± 5.2	63.2 ± 6.7
C17:0	28.4 ± 11.0	36.8 ± 15.1	38.9 ± 6.2 ^ab^	35.6 ± 14.4 ^a^	23.0 ± 10.0	29.7 ± 8.4
C18:0	27.3 ± 11.3 ^a^	32.7 ± 13.1 ^ab^	53.7 ± 17.2 ^ab^	22.9 ± 5.5	19.1 ± 8.4	20.6 ± 9.8
C20:0	17.9 ± 6.9	34.5 ± 33.1	28.2 ± 16.4	29.1 ± 8.0 ^a^	14.6 ± 13.3	19.2 ± 16.1
C21:0	43.9 ± 16.0 ^ab^	1.9 ± 3.8	16.7 ± 16.7	20.8 ± 18.6	9.6 ± 10.3	10.1 ± 15.7
C24:0	6.3 ± 11.0 ^ab^	11.4 ± 18.9	9.7 ± 14.5	ND	18.6 ± 14.1	18.3 ± 14.8
C14:1	24.8 ± 14.2 ^ab^	39.6 ± 37.6	30.0 ± 23.2 ^ab^	36.9 ± 15.6 ^ab^	8.0 ± 14.5	9.2 ± 16.6
C15:1	0.6 ± 1.8 ^ab^	6.7 ± 13.3	ND	ND	6.3 ± 11.8	7.6 ± 13.3
C16:1	31.7 ± 6.7	32.2 ± 21.2	46.5 ± 28.1	49.8 ± 7.3 ^ab^	35.2 ± 7.5	28.5 ± 9.3
C17:1	25.7 ± 23.4	18.3 ± 29.1	10.5 ± 11.6	16.7 ± 16.7	18.5 ± 22.6	16.7 ± 13.5
C18:1C	32.7 ± 10.4 ^ab^	43.4 ± 7.7 ^ab^	26.8 ± 6.1 ^ab^	39.2 ± 7.7 ^ab^	21.9 ± 4.6	17.8 ± 4.6
C18:2C	15.8 ± 7.2 ^ab^	45.2 ± 23.9	16.3 ± 7.5 ^ab^	27.0 ± 9.1	25.8 ± 4.8	28.3 ± 8.3
C18:3n6	14.2 ± 14.6	12.1 ± 12.5	19.2 ± 19.5	20.8 ± 13.8	22.2 ± 14.8	18.5 ± 15.4
C18:3n3	8.3 ± 5.4 ^ab^	21.6 ± 17.0	13.0 ± 10.0	14.9 ± 7.8	14.4 ± 9.0	16.6 ± 14.2
C20:1	7.2 ± 4.1	23.0 ± 18.7	5.2 ± 3.2	10.3 ± 3.2	7.8 ± 8.9	7.0 ± 7.9
C20:2	13.6 ± 19.4	14.9 ± 18.4	12.6 ± 21.8	49.5 ± 11.8 ^ab^	11.0 ± 8.3	9.5 ± 10.4
C20:3	14.4 ± 20.4	10.0 ± 13.3	ND	29.2 ± 18.2	9.7 ± 9.3	5.9 ± 9.4
C20:4	19.5 ± 28.9	9.1 ± 15.2	7.0 ± 5.0 ^ab^	28.8 ± 14.1	26.2 ± 9.9	30.1 ± 11.3
C20:5	14.3 ± 26.7	18.7 ± 23.3	13.2 ± 22.9	ND	14.3 ± 12.9	24.2 ± 19.2
C22:2	52.1 ± 22.9 ^ab^	10.0 ± 13.3	24.2 ± 34.6	31.1 ± 18.3 ^a^	3.3 ± 11.6	10.8 ± 58.4

ND: Not detected. Data are given as weight %. Values are average values ± standard deviation (Mean ± SD). a and b mean that significant differences were detected between groups formulas and colostrum, and formulas and milk, respectively. Abbreviations: OAF, oleic acid-based formula; PAF, palmitic acid-based formula; MCFAF, medium chain fatty acid formula-based formula; LAF, linoleic acid-based formula.

**Table 6:** Triacylglycerols (>0.1%) in piglet formulas, sow colostrum, and sow milk.

Fatty Acids	OAF	PAF	MCFAF	LAF	Colostrum	Milk
LaLaCy	0.4 ± 0.2 ^ab^	0.7 ± 0.5 ^ab^	2.4 ± 1.7 ^ab^	0.5 ± 0.2 ^ab^	ND	ND
CaCaM	0.4 ± 0.2 ^ab^	0.8 ± 0.6 ^ab^	2.4 ± 1.4 ^ab^	0.7 ± 0.5 ^ab^	ND	ND
LaLaCa	0.5 ± 0.3 ^ab^	0.8 ± 0.5 ^ab^	2.7 ± 1.6 ^ab^	0.5 ± 0.4 ^ab^	ND	ND
LaLaLa	2.5 ± 1.3 ^ab^	2.4 ± 2.0 ^ab^	8.1 ± 1.7 ^ab^	1.6 ± 0.7 ^ab^	ND	ND
OBuP	0.6 ± 0.3 ^ab^	1.4 ± 1.2 ^ab^	0.5 ± 0.3 ^ab^	0.4 ± 0.2 ^ab^	ND	ND
LaLaM	1.5 ± 0.8 ^ab^	1.6 ± 1.5 ^ab^	5.8 ± 2.0 ^ab^	1.1 ± 0.6 ^ab^	ND	ND
SPBu	0.6 ± 0.5 ^ab^	0.6 ± 0.5 ^ab^	2.3 ± 1.7 ^ab^	0.2 ± 0.1 ^ab^	ND	ND
LaLaP	0.6 ± 0.3 ^ab^	0.6 ± 0.6 ^ab^	2.0 ± 0.7 ^ab^	0.3 ± 0.2 ^ab^	ND	ND
MMLa	0.4 ± 0.2 ^ab^	0.6 ± 0.5 ^ab^	1.9 ± 1.0 ^ab^	0.3 ± 0.2 ^ab^	ND	ND
LLLn	0.5 ± 0.3 ^ab^	0.0 ± 0.0 ^ab^	0.6 ± 0.4 ^ab^	4.7 ± 1.9 ^ab^	1.1 ± 1.2	0.1 ± 0.2
SLaLa	0.6 ± 0.4 ^ab^	0.6 ± 0.4 ^ab^	1.1 ± 0.4 ^ab^	0.1 ± 0.0 ^ab^	ND	ND
PMLa	0.2 ± 0.2 ^ab^	0.4 ± 0.4 ^ab^	1.6 ± 0.3 ^ab^	0.3 ± 0.1 ^ab^	ND	ND
LLL	4.7 ± 1.7 ^b^	0.1 ± 0.2 ^a^	4.0 ± 1.4 ^b^	13.4 ± 2.4 ^ab^	5.7 ± 3.3	0.9 ± 1.2
LMP	0.3 ± 0.1 ^ab^	0.4 ± 0.3 ^ab^	0.3 ± 0.1 ^ab^	0.2 ± 0.0 ^ab^	0.7 ± 0.3	1.3 ± 0.6
OML	0.1 ± 0.1 ^ab^	0.1 ± 0.1 ^ab^	0.1 ± 0.1 ^ab^	0.1 ± 0.0 ^ab^	1.2 ± 0.3	1.5 ± 0.6
LPL	3.1 ± 0.6 ^ab^	1.1 ± 0.5 ^ab^	2.6 ± 0.8 ^ab^	7.8 ± 1.8 ^ab^	8.8 ± 3.9	4.5 ± 3.3
OLL	6.8 ± 1.6 ^b^	0.3 ± 0.2 ^ab^	4.9 ± 2.4 ^a^	11.2 ± 1.1 ^ab^	6.9 ± 1.8	2.4 ± 1.5
OPM	1.2 ± 0.2 ^a^	2.0 ± 0.9 ^a^	1.0 ± 0.1 ^a^	0.8 ± 0.1 ^b^	0.5 ± 0.3	1.5 ± 0.6
PPL	4.9 ± 3.1	7.7 ± 1.9 ^a^	3.5 ± 1.6 ^b^	2.3 ± 1.6 ^ab^	4.6 ± 1.4	6.3 ± 1.1
OPL	7.6 ± 1.4 ^ab^	6.7 ± 1.9 ^ab^	5.3 ± 0.9 ^ab^	7.9 ± 0.8 ^ab^	14.1 ± 2.6	14.3 ± 2.0
OOL	5.5 ± 1.5 ^ab^	0.9 ± 0.7 ^ab^	3.0 ± 1.4 ^ab^	4.6 ± 0.8 ^a^	6.7 ± 1.3	4.6 ± 1.2
SLL	0.6 ± 0.7 ^ab^	0.3 ± 0.2 ^ab^	1.1 ± 0.4 ^ab^	2.4 ± 0.3 ^b^	2.5 ± 0.5	1.7 ± 0.4
OPP	12.7 ± 2.5 ^ab^	21.3 ± 6.1 ^ab^	9.8 ± 3.3 ^a^	8.9 ± 0.6 ^a^	5.9 ± 2.4	8.7 ± 1.7
OOP	14.0 ± 2.6 ^ab^	15.7 ± 2.4 ^ab^	9.1 ± 1.1 ^ab^	9.7 ± 1.6 ^ab^	9.0 ± 2.4	12.7 ± 2.7
OOO	9.2 ± 3.4 ^ab^	3.6 ± 3.6	4.1 ± 2.9	5.7 ± 5.2	2.8 ± 0.9	3.3 ± 1.3
LSO	2.5 ± 1.6 ^ab^	2.3 ± 1.3 ^ab^	1.0 ± 0.6 ^ab^	1.9 ± 0.7 ^ab^	2.93 ± 0.88	3.41 ± 1.37
PPS	0.6 ± 0.4	1.1 ± 1.0	0.7 ± 0.4	0.4 ± 0.0 ^a^	0.5 ± 0.3	0.7 ± 0.9
SPO	3.6 ± 0.5 ^ab^	5.8 ± 0.7 ^a^	2.4 ± 0.6 ^b^	2.3 ± 0.5 ^b^	3.0 ± 1.5	5.9 ± 2.8
OOS	2.5 ± 1.2 ^ab^	2.4 ± 1.3 ^ab^	1.3 ± 0.2 ^ab^	1.2 ± 0.1 ^ab^	1.4 ± 0.6	2.4 ± 1.4
SPL	ND	ND	ND	ND	3.4 ± 0.9	4.8 ± 1.0
LLPo	ND	ND	ND	ND	1.2 ± 0.7	0.4 ± 0.3
LPLn	ND	ND	ND	ND	1.3 ± 0.8	0.4 ± 0.3
LPPo	ND	ND	ND	ND	2.3 ± 0.6	3.0 ± 1.3
OPoL	ND	ND	ND	ND	1.5 ± 0.7	0.8 ± 0.6
OOLn	ND	ND	ND	ND	1.6 ± 0.4	0.6 ± 0.4
PPoP	ND	ND	ND	ND	0.8 ± 0.5	2.3 ± 0.9
OPPo	ND	ND	ND	ND	3.3 ± 1.3	5.0 ± 0.9
OPoO	ND	ND	ND	ND	1.4 ± 0.4	1.5 ± 0.2
UPU	24.6 ± 3.9 ^ab^	23.4 ± 3.8 ^ab^	16.9 ± 2.5 ^ab^	25.3 ± 1.5 ^ab^	37.8 ± 3.7	38.0 ± 2.8
UPS	25.1 ± 3.5 ^ab^	41.3 ± 4.4 ^ab^	19.1 ± 4.7 ^b^	16.2 ± 1.9 ^b^	19.0 ± 6.0	31.4 ± 5.3
SPS	6.8 ± 2.2 ^ab^	11.9 ± 5.2 ^ab^	10.5 ± 3.1 ^ab^	3.4 ± 0.6 ^a^	1.2 ± 0.7	1.9 ± 1.6

ND: Not detected. Data are given as weight %. Values are average values ± standard deviation (Mean ± SD). a and b mean that significant differences (*p* < 0.05) were detected between groups formula and colostrum, and formula and milk, respectively. There are different isomers of these triacylglycerols, e.g., LPL, OPL, OOP, and OOS. The structural isomer was calculated according to the ratio of abundances of fragments generated by loss of sn-1, sn-2, and sn-3. The results showed sn-L/P/L, sn-O/P/O, sn-O/P/L, and sn-O/S/O were the main form in sow milk fats, while sn-L/L/P, sn-O/O/P, sn-L/O/P, and sn-OOS were dominant in formula fats [12,13,31,32]. Abbreviations: OAF, oleic acid-based formula; PAF, palmitic acid-based formula; MCFAF, medium chain fatty acid formula-based formula; LAF, Linoleic acid-based formula; Bu, C4:0; Cy, C8:0; Ca, C10:0; La, C12:0; M, C14:0; P, C16:0; Po, C16:1; S, C18:0; L, C18:1; O, C18:2; Ln, C18:3; U, unsaturated fatty acid; S, saturated fatty acid.

## Data Availability

The data presented in this study are available on request from the corresponding author.
